# Monitoring of Hydrogen Emission from Bacteria in Food, Animals and in the Blood of Humans Suffering from Lyme Disease by A Specific Hydrogen Sensor

**DOI:** 10.3390/antibiotics9070427

**Published:** 2020-07-21

**Authors:** Bruno Kolb, Lorina Riesterer, Anna-Maria Widenhorn, Leona Bier

**Affiliations:** Student Research Centre SFZ, D-88662 Überlingen, Germany; lorinariesterer99@gmail.com (L.R.); A-M.Widenhorn@web.de (A.-M.W.); leona.bier@gmail.com (L.B.)

**Keywords:** bacteria, food, Lyme disease, antibiotics, antibiotic resistance, garlic, hydrogen sensor, headspace gas chromatography

## Abstract

A novel straightforward analytical technique was developed to monitor the emission of hydrogen from anaerobic bacteria cultured in sealed headspace vials using a specific hydrogen sensor. The results were compared with headspace gas chromatography carried out in parallel. This technique was also applied to investigate the efficacy of chemical antibiotics and of natural compounds with antimicrobial properties. Antibiotics added to the sample cultures are apparently effective if the emission of hydrogen is suppressed, or if not, are either ineffective or the related bacteria are even resistant. The sensor approach was applied to prove bacterial contamination in food, animals, medical specimens and in ticks infected by *Borrelia* bacteria and their transfer to humans, thus causing Lyme disease. It is a unique advantage that the progress of an antibiotic therapy can be examined until the emission of hydrogen is finished. The described technique cannot identify the related bacteria but enables bacterial contamination by hydrogen emitting anaerobes to be recognized. The samples are incubated with the proper culture broth in closed septum vials which remain closed during the whole process. The personnel in the lab never come into contact with pathogens and thus safety regulations are guaranteed.

## 1. Introduction

Infections by pathogenic microbes are a growing threat to public healthcare today. Bacterial infections of humans may be caused by contaminated food of animal or vegetable origin, by contact with infected animals or through the bite of an insect. Bacteria emit volatile compounds which can be analyzed by various techniques of gas analysis, preferably gas chromatography. Bacteria specimens are usually cultured by the common plate-based assays technique. The analysis of emitted volatiles requires closed containers, however, and the technique of static headspace gas chromatography (HS-GC) is therefore the preferred technique since the samples are cultured in closed crimp capped headspace vials. This technique was originally applied in 1965 for examining the growth of bacteria in milk [1]. The emitted volatiles comprise a wide range of organic and inorganic compounds [2]. The unique fingerprint pattern of the emitted volatile organic compounds (VOCs) in a headspace chromatogram was used to identify the relevant bacteria on the genus or even the species level [3,4,5]. On the other hand, it might be useful to take just a single compound as the characteristic emission of bacterial contamination. In this work, hydrogen (H_2_) was used as such for the leading compound. Although the single analysis of emitted H_2_ is not sufficient to identify the relevant bacterium on the genus or species level, it can classify its type: obligate anaerobes emit hydrogen together with a mixture of VOCs, but need an oxygen free condition which can be achieved by replacing the ambient air in the vial by flushing with nitrogen. As such, a headspace vial under nitrogen is thus used as a mini-anaerobic chamber in which the bacteria can thrive. Obligate aerobes generate carbon dioxide and water, but no hydrogen, and the emission of CO_2_ with the lack of hydrogen indicates the presence of such an obligate aerobe. Facultative anaerobes emit H_2_ and CO_2_ and some organic compounds, e.g., ethanol. The proof of emitted H_2_, therefore, was taken in this work to recognize bacterial contamination in food and medical specimens and can be applied particularly to screening applications without the ambition to identify the relevant bacteria which may then be performed subsequently with proven methods. Such an application by HS-GC for proving bacterial contamination in vegetables, animal food and medical specimens has been described [6]. In addition, the contamination of ticks by *Borrelia* and their transfer to humans causing Lyme disease [7] has been investigated. Gas chromatography is an efficient technique to separate various components in a complex mixture, but if a single compound only should be detected, a specific sensor may be sufficient. Specific tailor-made biosensors have been applied to identify specific bacteria [8]. In this work, however, we use a specific hydrogen sensor to monitor H_2_ as the leading volatile emission for all of the anaerobic bacteria that are responsible for the bacterial contamination. The proof of H_2_ comprises all anaerobes: obligate anaerobic, facultative anaerobic and microaerophilic bacteria. The potential of the new sensor approach is presented in this work by comparing it with the result of the HS-GC analysis carried out in parallel.

Another application of the H_2_ sensor approach described in this study is the proof of bacterial infection in ticks, and the following diagnosis and therapy of the resulting tick-borne Lyme disease of an infected individual. Again, the H_2_ sensor approach does not enable the identification of a single species, such as the responsible *Borrelia burgdorferi* and the various *Borrelia* species and subspecies, but has the advantage that the H_2_ response comprises the whole complex of co-infection by microbes with similar pathogenic characteristics, because Lyme disease is always associated with multiple microbes [9]. The complex mixtures of all those responsible pathogens have been summarized as *Borreliella* [7,10], but since this is controversially discussed [11] this complex mixture of all possible co-infections should therefore be described here as *Borrelia*+. The list of possible coinfections includes *Chlamydia*, *Bartonella*, *Mycoplasma*, *Babesia*, *Anaplasma*, *Ehrlichia*, *Rickettsia* and several more. These co-infections have important clinical, diagnostic, and therapeutic implications since co-infected humans tend to display a more severe manifestation of Lyme disease. The proof of H_2_ therefore enables a broad range of diagnosis and therapy of the most important known or putative tick-borne pathogens, not only in vitro but more importantly even in the blood of an infected patient. Such an in vivo diagnosis is also presented in this article. Here, it is important to note that not only can bacterial infection be recognized by the H_2_ sensor approach but, moreover, the effect of antibiotics against the responsible microbes, if added to the samples in the vials, is also detected. Antimicrobial resistance is a serious and current issue in medicine. Infections caused by pathogenic microbes are a growing threat to public healthcare today if treated by an ineffective therapy due to resistance against antibiotics [12]. For this reason, novel antibiotics are needed to replace such multi-resistant chemical antibiotics and there is growing interest in searching for natural compounds with biocidal properties. A few examples from some natural compounds (not all shown here) are selected just to demonstrate the potential of the H_2_ approach to prove bacterial contamination in food, in animals and medical specimens, and to test the efficacy of natural and chemical antibiotics.

## 2. Results

### 2.1. Interpretation of the Chromatograms

**Figure 1:**
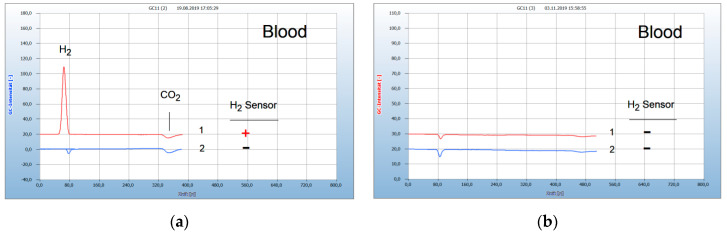
Analysis of blood after garlic therapy by in vivo test. Gas chromatography: column Chromosorb 102, carrier gas ambient air, 6 mL headspace vials, 2.5 mL culture broth, blank: cotton bud in culture broth, H_2_ emission (**+**), no H_2_ emission (**−**). (**a**) 1 = blood after insufficient doxycyclin theray (**+**), 2 = blank: (**−**). (**b**): 1 = blood after 4 weeks garlic therapy (**−**), 2 = blank: (**−**).

A simple gas chromatograph for students was used for this work. The column was not thermostatted but operated at room temperature with ambient air as the carrier gas and using a heat conductivity detector. Depending on the differences in heat conductivity, both positive and negative peaks are generated. Hydrogen, due to its high heat conductivity produces a positive peak with good sensitivity while carbon dioxide, due to its lower heat conductivity, causes a negative peak at the end of the chromatogram. Two negative peaks are shown in the gas chromatograms, one close to the rear of the positive hydrogen peak and later at the end of the chromatograms. These negative peaks can be of different origin. The small negative peak at the rear of the hydrogen peak corresponds to the retention time of air, but it is caused by a deficit of oxygen in the air in the headspace of the vials by various oxidation processes. Facultative anaerobes in the closed vials start with oxidative metabolism with O_2_ consumption and CO_2_ generation, but move to fermentation with H_2_ emission due to declining O_2_ concentration in the vial, and a CO_2_ peak at the end of the chromatograms is thus generated. Such a negative CO_2_ peak may also be generated by aerobes in the sample, since obligate aerobes produce CO_2_ and water, and O_2_ from the air in the headspace is thus transferred to CO_2_. The headspace gas therefore is enriched with nitrogen and due to the lower heat conductivity, compared with the carrier gas, the air causes the small negative peak at the rear of the positive H_2_ peak. An additional source of such a negative CO_2_ peak is caused by blood samples in the headspace vials because blood is still active and converts the oxygen of the air in the vials to CO_2_. These negative CO_2_ peaks are therefore not unambiguously significant for bacterial activity contrary to H_2_, which is used here as the leading compound for all active anaerobic bacteria. The negative peak from the O_2_ deficit is often hidden beneath the hydrogen peak with the Silicagel column while a column with Chromosorb 102 separates it better but has a longer retention time for CO_2_. Both column types are used for the various examples presented here. Since the gas chromatography (GC) columns with the cheap GC student instrument are operated always at varying room temperatures the retention times of the peaks may vary slightly too (e.g., Figure 1). Explanation of the chromatograms: the *x*-axis extends from 0 to 800 s, *y*-axis gas phase concentration in arbitrary units.

### 2.2. Method

The samples are placed into headspace vials, which are then sealed by a crimp closure. The 6 mL vials already contain 2.5 mL of sterile culture broth. Solid samples are cut into small pieces. The ball of cotton wool taken from the cotton bud used to collect a blood or smear sample is also inserted into the liquid culture broth. Blood samples were collected by piercing a fingertip with a needle, except the example given in Figure 1, where the blood sample was withdrawn from the vein by a physician. The resulting droplet of blood (~50 µL) from the fingertip was sucked into a sterile cotton ball from a cotton bud in such a way that the cotton ball did not come into contact with the skin. The blood-soaked cotton ball was then inserted into the sterile culture broth in the vial which was closed immediately by a crimp closure. Blood samples were collected exclusively from the author B.K. and from member of his family. All subjects gave their consent before they participated in this study.

**Figure 2:**
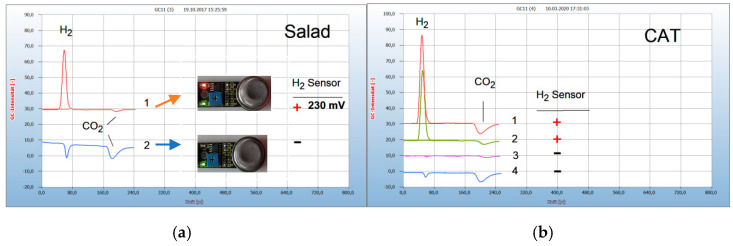
Obligate anaerobes, facultative anaerobes, and obligate aerobes by headspace gas chromatography (HS-GC) and the H_2_ sensor approach. Gas chromatography: column Silicagel, carrier gas ambient air. H_2_ emission (**+**), no H_2_ emission (**−**). (**a**) Chopped salad leaves in 20 mL headspace vials, no culture broth; culture time 15 h; 1 = salad packed in plastic container with facultative anaerobes (**+**), 2 = salad unpacked with aerobes (**−**). (**b**) Feces from a cat in a 6 mL headspace vial with 2.5 mL culture broth; 1 = feces with obligate anaerobes (H_2_) and aerobes (CO_2_) under headspace gas N_2_ (**+**), 2 = added amoxicillin (**+**), 3 = added oil of cloves (**−**), 4 = feces under headspace gas air (**−**) with aerobes (CO_2_).

Bulky samples, such as the salad samples (cf. Figure 2a), were placed into 20 mL vials as chopped solid leaves without culture broth. Ticks were not crushed but placed intact in the culture broth. The charged vials were then cultured at 35 °C but the necessary time depends on the reproduction rate of the relevant bacteria. In general, after 12 h a measurable H_2_ peak can be obtained [6]. Some bacteria, e.g., spirochetes, were cultured for three days due to their slow reproduction rate. After, a 0.3 mL aliquot of the headspace gas was withdrawn by a gas tight syringe by piercing the septum of the vial and injecting it into the gas chromatograph whilst also flushing it against the H_2_ sensor. The presence of H_2_ is indicated with that particular sensor by a red light, though in addition to this optical response, the electrical signal can also be traced by measuring the voltage output (mV). Several chemical and natural antibiotics were tested and a few milligrams of solid antibiotics are added to the culture broth or one microliter for liquid antibiotics. In the following examples, the GC analysis is compared with the response from the H_2_ sensor and the result is included in the chromatograms either by the positive (**+**) or the missing (**−**) H_2_ red light response.

### 2.3. Proof of Aerobes and Anaerobes by HS-GC and by the H_2_ Sensor Approach

Hydrogen released from bacteria was analyzed by a specific hydrogen sensor and the results compared with those from static HS-GC. The procedure is shown in Figure 2a.

Figure 2a gives an example of two mixed salad samples collected from a supermarket. The salad sample is shown as no. 1 in Figure 2a and was packed in a closed plastic container. Such salads are often found contaminated by *Salmonella* bacteria, generated by the wet juice from the chopped salad and this apparently was also the case here as shown by the H_2_ peak in the chromatogram. The H_2_ sensor confirmed this result by the red light and also by measuring the voltage output of 230 mV. The other salad sample, no. 2 in Figure 2a, was unpacked and offered unpacked as bio-salad and produced neither an H_2_ peak in the chromatogram nor a sensor signal. The two negative peaks of CO_2_ in this chromatogram apparently are generated by obligate aerobes. The determination of obligate anaerobes is shown in Figure 2b. Feces from carnivores contain obligate anaerobes, e.g., *Clostridia, Escherichia coli*. Small pieces from the feces from a cat were cultivated in the culture medium in 6 mL headspace vials and the air was replaced by flushing with nitrogen to provide the necessary oxygen-free gas phase. The resulting H_2_ peak in the chromatogram no. 1 corresponded with the positive sensor signal (**+**). This example shows also the possibility to test the efficacy of antibiotics. A few milligrams of added amoxicillin to the charged vial had no effect, as shown by the same H_2_ peak (no. 2) and the positive H_2_ sensor signal (**+**), while a small droplet from the oil of the clove apparently killed the anaerobes (no. 3), as shown by the missing H_2_ peak and the negative sensor signal (−). Oil of cloves was already found as a very effective natural antibiotic [7,12]. Normal air in the vials prevented the generation and emission of H_2_ by obligate anaerobes due to the presence of oxygen (no. 4).

### 2.4. Analysis of Food

**Figure 3:**
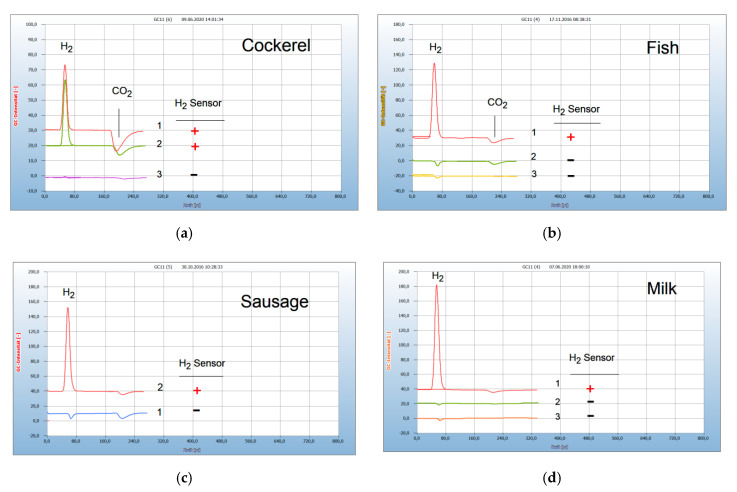
Bacterial contamination in food, analyzed by HS-GC and the H_2_ sensor approach. Gas chromatography: column Silicagel, carrier gas ambient air, 6 mL headspace vials, 2.5 mL culture broth. H_2_ emission (**+**), no H_2_ emission (**−**). Blank: cotton bud in culture broth. (**a**) 1 = meat from a cockerel (**+**), 2 = added penicillin (**+**), 3 = added oil of cloves. (**b**) 1 = meat from salmon from supermarket, (**+**), 2 = trout freshly caught from a clear river (**−**), 3 = blank (**−**). (**c**) 1 = liver-sausage, fresh (**−**), 2 = after 8 days in a refrigerator (**+**). (**d**) 1 = raw milk, 1 mL in 2.5 mL culture broth (**+**), 2 = pasteurized milk, 1 mL in 2.5 mL culture broth (**−**), 3 = blank (**−**).

Food poisoning can occur when disease-causing bacteria spread to food and are consumed. Food producing animals from fish and poultry farming are often contaminated by microbes and are therefore treated by antibiotics, but an excess application can cause antibacterial resistance. There is serious concern regarding the increasing number of multi-resistant chemical antibiotics, and the search for natural antibiotics as an alternative to chemical antibiotics is of increasing interest. Two such examples are shown in Figure 3a,b. Meat from a cockerel (no. 1 in Figure 3a) purchased from a supermarket was contaminated by facultative anaerobes, as indicated by the H_2_ peak and also by aerobes by the CO_2_ peak. The addition of penicillin (no. 2 in Figure 3a) to the culture broth in the sample vial was ineffective, or the responsible bacteria were already resistant, because the H_2_ emission was still active. The oil of the cloves (no. 3 in Figure 3a) has eradicated all bacteria responsible for the emission of both H_2_ and CO_2_. Salmon (Figure 3b) from an aquatic farm and purchased from a supermarket was contaminated by facultative anaerobes, as shown by the H_2_ peak, and also by aerobes as indicated by the CO_2_ peak (no. 1). A freshly caught trout from a clean river generated no H_2_ emission and was thus not contaminated by anaerobes, but still contained some aerobes as shown by the CO_2_ peak (no. 2). The chromatogram from the blank sample (no. 3) showed neither H_2_ nor CO_2_ peaks.

The next example in Figure 3c shows the possibility to control the stability of the food. The liver-sausage was spoiled already after one week stored, even in a refrigerator (no. 2), as indicated by the H_2_ emission compared with the fresh quality (no. 1). The example in Figure 3d presents a difference between raw and pasteurized milk. Pasteurization apparently eliminates all microbes found in raw milk.

### 2.5. Application for Lyme Disease

**Figure 4:**
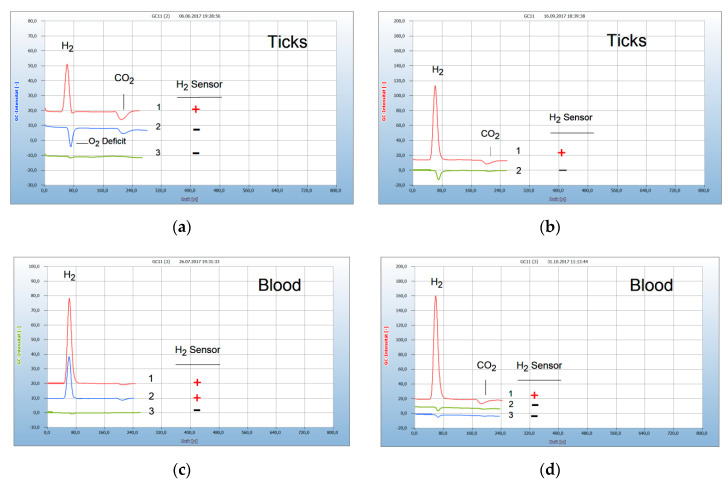
Infected ticks and transfer to blood by in vitro tests. Gas chromatography: column Silicagel, carrier gas ambient air, 6 mL headspace vials, 2.5 mL culture broth. H_2_ emission (**+**), no H_2_ emission (**−**). Blank: cotton bud in culture broth. (**a**) 1 = tick, infected by *Borrelia+* (**+**), 2 = tick, infected by aerobes (**−**), 3 = blank (**−**). (**b**) 1 = tick, infected by *Borrelia+* (**+**), 2 = tick, not infected (**−**). (**c**) 1 = blood, infected by *Borrelia+* (**+**), 2 = tick, infected and responsible for bacteria transfer (**+**), 3 = blank (**−**). (**d**) 1 = blood, infected by *Borrelia+* (**+**), 2 = healthy blood (**−**), 3 = blank (**−**).

People may not only be infected by food-borne microorganisms, but also through contact with animals carrying pathogenic parasites. People who spend time in grassy and wooded environments are at an increased risk of exposure to catching ticks which can cause Lyme disease. Ticks are not only contaminated by *Borrelia burgdorferi* and its subspecies but can also carry further co-infected pathogens with similar characteristics. Emitted H_2_ is the common metabolite from this tick-borne mixture comprising all facultative anaerobic subspecies and similar pathogens. Such a single guarding compound simplifies diagnosis and the search for a suitable therapy. However, not all ticks in an infected area are contaminated, therefore, after a tick bite the removed tick should be tested prior to medical treatment. The chromatograms in Figure 4a present the result from two ticks, one contaminated and the other not. The resulting H_2_ peak from the infected tick (no. 1) corresponds with the positive sensor signal (**+**) and also the missing H_2_ peak (no. 2) with the negative sensor signal (**−**).

Figure 1 presents the result from a person bitten by a tick and describes the complete course of infection up to a successful therapy. After having been bitten by a tick a red “erythema chronicum migrans” appeared at the upper leg and the physician prescribed the usual doxycyclin therapy. A blood sample was taken from the vein and a serological test confirmed an infection by *Borrelia sensu lato*. However, even after 3 weeks treatment the patient still complained about typical pains and a blood sample analyzed now by the HS-GC technique shows in fact still a remarkable H_2_ peak (no. 1 in Figure 1a) and a positive sensor signal (**+**) present. The doxycyclin therapy, with a daily dose of 200 mg, apparently was not sufficient since most experts recommend at least 400 mg/day. Since the patient could not endure this antibiotic, the therapy was continued by a daily dose of 900 mg garlic powder from garlic capsules. After 4 weeks treatment, the blood no longer developed H_2_ (no. 1 in Figure 1b) and the garlic therapy apparently was successful. A repeated control analysis one month later confirmed this result since no re-growth occurred. Garlic was used here successfully as an alternative antibiotic. However, the bacteria may have migrated into hidden parts as an immobile stationary phase and can change back to the motile helical form. Such a re-growth was described [7] from a patient who was first cured of Lyme disease by the standard doxycyclin therapy, but H_2_ was emitted again by *Borrelia+* after a virus infection, probably due to the weakened immune system, but could then be cured by the garlic therapy.

## 3. Discussion

A new method is presented to prove bacterial infection in food, animals and humans using the emission of hydrogen. Bacterial contamination was detected in food (salad, milk, liver-sausage) and in the meat of animals (cockerel, fish, cat). Food, particularly offered in supermarkets, is often contaminated by microbes and requires careful control to protect the consumers from food-borne diseases. The H_2_ approach can also be used to study the efficacy of natural and chemical antibiotics. We have investigated a limited number of natural antibiotics, such as seed of cress, teasel, propolis, tree resin and also some essential oils, but with varying and not unambiguous success, depending on the sample type, the relevant bacteria and some other parameters. From these compounds, only garlic (*Allium sativum*) and oil of cloves (*Eugenia caryophyllus*) have emerged as universally applicable natural antibiotics [6,7] and two such examples are presented here. The addition of the oil of cloves to the contaminated meat of cockerel (Figure 3a) in the cultured samples eliminated the bacterial contamination contrary to penicillin.

The other antibiotic favorite was garlic, administered as capsules for this study, which are more convenient and above all less unpleasant than rough garlic [6]. Garlic was applied against *Borrelia*+ in infected ticks and in the blood of patients suffering from tick-borne Lyme disease. The antibacterial effect of garlic had already been found by Louis Pasteur and also described in detail [13]. The Swedish army has applied such garlic capsules successfully as tick-repellents for military personnel [14]. The oil of garlic was found effective in vitro tests against stationary phase *Borrelia burgdorferi* together with many other essential oils [15,16]. The proof of contamination by *Borrelia* bacteria in ticks and their transfer to the blood of a bitten human causing Lyme disease is of particular interest, since Lyme disease is a potentially fatal illness spread through the bite of ticks that carry infected bacteria, predominantly *Borrelia* species, including *Borrelia burgdorferi* and transmitted to the bloodstream of humans. This tick-borne illness comprises serious health effects such as joint malfunctions and neurological or cardiac disorders and many others. If, in its early stages, pathological skin lesions (namely *erythema migrans*) appear, such an infection is obvious, but often these lesions are not visible and so the infection is therefore ignored with fatal consequences. Even if the *erythema migrans* appears late, sometimes after weeks or months, this may be too late to start an effective therapy because Lyme disease patients can be cured successfully only in the early stage of infection with the common 2–4 weeks antibiotic treatment. If, however, an infection is not recognized due to missing skin lesions, Lyme disease progresses, and the patient continues to suffer from chronic symptoms described as post-treatment Lyme disease syndrome and this persistent borreliosis is no longer accessible by the common antibiotics. It is therefore important to start the antibiotic therapy in due time and this requires a reliable diagnosis as soon as possible after a tick bite. The available techniques of diagnosis, such as ELISA and Western Blot are not always sufficiently reliable to make a definite diagnosis. This is because these serological assays are indirect tests since they measure an antibody’s response to the infection, not the infection itself, and these need 2 to 6 weeks after infection while the efficacy of antibiotic treatment declines already after 4 weeks [17]. All diagnostic devices available bear major drawbacks in terms of sensitivity and specificity [18]. Moreover, these serological tests are not standardized and antibodies, once generated, may still remain in blood even if the bacteria are already eradicated. The same argument applies for the PCR technique which identifies residual DNA. A positive PCR test almost always guarantees Lyme infection, but there can be false-negative results if executed in blood either too early or too late. All these indirect diagnostic techniques cannot control the progress of an antibiotic therapy, and it is for these reasons that the recommendations on the necessary dosage and the adequate length of therapy vary widely from one week up to several months between many experts due to the uncertainty of the applied therapy. An antibiotic therapy with too long an extension may damage the microbiome and on the other hand an interruption that is too early is particularly dangerous. For all these reasons, a reliable direct proof of pathogenic bacteria is urgently needed and, as such, an approach is offered in this study with the H_2_ sensor technique. The emission of H_2_ from an infected blood sample is a direct proof of the bacterial infection. As Lyme disease is always associated with the complex mixture of co-infections, it is particularly interesting for a patient that monitoring the H_2_ emission allows the progress of a suitable therapy to be controlled until those pathogenic microbes are eradicated altogether, as indicated by the disappearance of the H_2_ signal. Finally, a patient is less interested in knowing the exact composition of the responsible pathogenic mixture, if an efficient therapy eradicates them all, together with the accompanying pain. However, a natural compound with a biocide property found by an in vitro test of a blood sample in a headspace vial may not necessarily be as effective if administered in vivo to a patient suffering from borreliosis, since such an in vivo trial is hardly to be executed as a patient will certainly prefer to rely on proven chemical antibiotics, e.g., doxcycyclin rather than to act as an experimental volunteer for suspected antibiotics. Despite this problem, such an in vivo result is presented in this work, where garlic was found to be effective in curing Lyme disease even after an insufficient doxycyclin therapy.

## 4. Conclusions

The H_2_ approach has the unique advantage that the whole procedure is carried out in hermetically closed vials, starting with sample preparation. When the samples are inserted into the culture broth, the headspace vials are capped with PTFE-lined septa and sealed with a crimped closure. The septum remains tight and several gas samples can be analyzed at given time intervals, thus enabling kinetic measurements. After analysis, the closed vials with their contents can be sterilized at the necessary high temperature and then safely discarded. Thus, the personnel in a lab never come into contact with the pathogens. Both techniques, HS-GC and the H_2_ sensor approach, may have different advantages and applications. HS-GC can be carried out with commercially available automated instruments and, thus, up to 100 samples can be processed unattended. It is therefore ideal for a broad range of screening purposes. The H_2_ sensor approach on the other hand is a simple technique and since such a H_2_ sensor is a very cheap device it is feasible that the necessary analyses to determine Lyme disease, including controlling the progress of a therapy, may be carried out in the office of a physician or even in a pharmacy since no strict safety requirements are afforded.

Both techniques of HS-GC and the H_2_ sensor approach are perfectly suited for quantitative analysis since the resulting peak area of an HS-GC analysis and the electrical output (mV) of an H_2_ sensor lend themselves to computer calculation. The real problem for quantitative determination, however, is the preparation of certified calibration standards and this aspect needs further research efforts. Finally, it should be admitted that all examples presented in this article should only be taken as suggestions to encourage more detailed research in the future, specifically to evaluate the proposed method of H_2_ emission, because our research project is finished now and we have no further possibility to continue.

## 5. Materials and Instrumentation

Gas chromatograph GC-AK 11, Aug. Hedinger GmbH & Co KG, Stuttgart, Germany; GC-columns: 0.8 m × 6 mm polyamide tube, packed with Silicagel 60/80 mesh and 0.8 m × 6 mm polyamide tube, packed with Chromosorb 102, 60/80 mesh. Headspace vials: 6 mL and 20 mL crimp-capped with PTFE-laminated butyl rubber septa from PerkinElmer LAS (Germany) GmbH, Rodgau, Germany. Culture broth: CASO-Bouillon, Merck Life Science GmbH, Darmstadt, Germany; vendor: Mibius e.K. Düsseldorf, Germany, tryptic soy broth acc EP+USP 3080r-20p; composition: pancreatic digest of casein, 17 g; papaic digest of soya bean meal, 3 g; sodium chloride, 5 g; dipotassium hydrogen phosphate, 2.5 g; glucose monohydrate. Garlic capsules with pulverized garlic, 500 mg/tablet, 1 mg Allicin, Hirundo Products, FL-9493 Mauren. Hydrogen sensor: Keyes MQ-8 Hydrogen gas sensor, Modul KS-046, fluxworkshop, purchased from eBay.

## References

[1] Bassette R., Claydon T.J. (1965). Characterization of some bacteria by gas chromatographic analysis of head space vapors from milk cultures. J. Dairy Sci..

[2] Kolb B., Ettre L.S. (2006). Static Headspace-Gas Chromatography: Theory and Practice.

[3] Seifert S., Böhnel H., Gierke S., Heine A., Hoffman D., Sukop U., Boege D. (1986). Identification of anaerobic bacteria by fatty acid pattern recognition. Int. Lab..

[4] Heiterfuß S., Heine A., Seifert S. (1990). Identification of anaerobic bacteria by determination of non-volatile dicaboxylic acids after derivatization. J. Chromatog. Biomed. Appl..

[5] Tait E., Perry J.D., Stanforth S.P., Dean J.R. (2014). Identification of volatile organic compounds produced by bacteria using HS-SPME-GC-MS. J. Chromatogr. Sci..

[6] Kolb B., Riestere L., Bier L., Widenhorn A.-M. (2019). Proof of bacteria and the activity of chemical and natural antibiotics by headspace gas chromatography. J. Anal. Sci. Technol..

[7] Kolb B., Schneider E.M., Riesterer L., Bier L., Hein T. (2019). Metabolic Profiling of *Borrelia* in Ticks and in Whole Blood Samples by Headspace Gas Chromatography—A Case Report. Am. J. Biomed. Sci. Res..

[8] Alahi M.E.E., Mukhopadhyay S.C. (2017). Detection Methodologies for Pathogen and Toxins: A Review. Sensors.

[9] Lantos P.M., Wormser G.P. (2014). Chronic coinfections in patients diagnosed with chronic Lyme disease: A systematic review. Am. J. Med..

[10] Adeolu M., Gupta R.S. (2014). A phylogenomic and molecular marker based proposal for the division of the genus *Borrelia* into two genera: The emended genus *Borrelia* containing only the members of the relapsing fever *Borrelia*, and the genus *Borreliella gen. nov.* containing the members of the Lyme disease *Borrelia* (*Borrelia burgdorferi* sensu lato complex). Antonie Van Leeuwenhoek.

[11] Winslow C., Coburn J. (2019). Recent discoveries and advancements in research on the Lyme disease spirochete *Borrelia burgdorferi*. F1000Research.

[12] Patini R., Mangino G., Martellacci L., Quaranta G., Masucci L., Gallenzi P. (2020). The Effect of Different Antibiotic Regimens on Bacterial Resistance: A Systematic Review. Antibiotics.

[13] Harris J.C., Cottrell S.L., Plummer D., Lloyd D. (2001). Antimicrobial properties of Allium sativum (garlic). Appl. Microbiol. Biotechnol..

[14] Stjernberg L., Berglund J. (2000). Garlic as an insect repellent. JAMA.

[15] Feng J., Zhang S., Shi W., Zubcevik N., Miklossy J., Zhang Y. (2017). Selective Essential Oils from Spice or Culinary Herbs Have High Activity against Stationary Phase and Biofilm Borrelia burgdorferi. Front. Med..

[16] Feng J., Shi W., Miklossy J., Tauxe G.M., McMeniman C.J., Zhang Y. (2018). Identification of Essential Oils with Strong Activity against Stationary Phase Borrelia burgdorferi. Antibiotics.

[17] Diagnostik und Therapie der Lyme-Borreliose, Leitlinien. Deutsche Borreliose-Gesellschaft.e.V.Jena.

[18] Marques A.R. (2015). Laboratory diagnosis of Lyme disease: Advances and challenges. Infect. Dis. Clin..

